# Comparison of quantitative computed tomography analysis and single-indicator thermodilution to measure pulmonary edema in patients with acute respiratory distress syndrome

**DOI:** 10.1186/1475-925X-13-30

**Published:** 2014-03-13

**Authors:** Fan Zhang, Chen Li, Jian-ning Zhang, Hai-peng Guo, Da-wei Wu

**Affiliations:** 1 Department of Critical Care Medicine, Qilu Hospital of Shandong University, Jinan, China

**Keywords:** Acute respiratory distress syndrome, Pulmonary edema, Quantitative CT analysis, Single indicator thermodilution method, Extravascular lung water

## Abstract

**Objective:**

To compare quantitative computed tomography (CT) analysis and single-indicator thermodilution to measure pulmonary edema in patients with acute respiratory distress syndrome (ARDS).

**Method:**

Ten patients with ARDS were included. All underwent spiral CT of the thorax for estimating gas content of lung (GV_CT_), tissue volume of lung (TV_CT_), tissue volume index (TVI), mean radiographic attenuation (CTmean) for the whole lung and gas-to-tissue ratio (g/t). Pulmonary thermal volume (PTV) and extravascular lung water index (ELWI) were determined by the PiCCO *plus* system. CT or single-indicator thermodilution variables were correlated with respiratory system compliance (Crs), PaO_2_/FiO_2_, and Acute Physiology And Chronic Health EvaluationII (APACHE II) and Sequential Organ Failure Assessment (SOFA) scores.

**Results:**

1) TV_CT_ and PTV were positively correlated (*r* =0.8878; *P* = 0.0006; equation of regression line: PTV = 1.0793 × TV_CT_ + 179.8) as were TVI and ELWI (*r* =0.9459; *P* < 0.0001; equation of regression line: ELWI = 1.4506 × TVI-8.7792). The bias between TV_CT_ and PTV as well as TVI and ELWI was -277 ± 217 and 0.62 ± 4.56, respectively. 2) ELWI and CT distribution of lung-tissue compartments were not correlated. 3) CT or single-indicator thermodilution variables were not correlated with Crs, PaO2/FiO2 or APACHE II or SOFA score.

**Conclusion:**

Quantitative CT analysis and single-indicator thermodilution showed good agreement in measuring pulmonary edema.

## Introduction

Interstitial lung edema is the most important pathological character of acute lung injury (ALI) and acute respiratory distress syndrome (ARDS) [1,2]. Quantitative measurement of lung edema can provide a useful marker of disease severity and prognosis [3,4]. Quantitative CT analysis and single-indicator thermodilution have been used to quantify lung edema clinically and experimentally.

Quantitative CT analysis provides a measure of lung volume, gas content (GV_CT_) and tissue volume (TV_CT_) [5]. It also helps divide the whole lung into 4 compartments by radiographic attenuation values of normally aerated, poorly aerated, nonaerated, and hyperinflated [5]. Although a classification of lung regions according to CT density is often used, various lung components (tissue, vessel, blood, edema) cannot be differentiated by radiologic density [6-11]. Moreover, radiation exposure, the risk of transferring critically ill patients, the relative high costs, and restricted availability all limit obtaining repeated measurements, so CT is not an ideal method to follow the clinical evolution of lung injury.

Extravascular lung water (EVLW) has been widely used to quantify pulmonary edema and guide fluid resuscitation in critically ill patients [12]. Single-indicator thermodilution is a relatively simple and safe method for repeatedly measuring EVLW. Unfortunately, many factors that may affect the accuracy of the single-indicator thermodilution method include vascular obstruction by emboli, thrombosis, severe hypoxic vasoconstriction, and, possibly, high airway pressure, which are common in patients with ALI/ARDS [13-22].

The gravimetric method of estimating EVLW is an experimental method and is the gold standard; it can be used to compare the wet and dry weight of the lung in animals or humans on autopsy, for only one measurement. The accuracy of the single-indicator method in measuring lung water has been confirmed by the gravimetric method in animals and human studies [23-26]. In an experimental animal study, quantitative CT analysis showed good agreement with the gravimetric method [27].

Measuring lung edema by the thermal indocyanine green-dye double-dilution method has shown good agreement with that by quantitative CT [28]. However, in mainland China, the single-indicator thermodilution method is used more than the thermal indocyanine green-dye double-dilution method. Therefore, we aimed to compare quantitative CT analysis and single-indicator thermodilution for diagnosis measurement of pulmonary edema in patients with ARDS.

## Materials and methods

### Patients

From March to December 2010, we included 10 consecutive patients with ARDS according to the criteria from the European American Consensus Conference on ARDS [1]. Patients had been admitted to the intensive care unit (ICU) of Qilu Hospital, Shandong University, and had undergone lung CT and PiCCO *plus* system measurement for clinical monitoring. We excluded patients who were < 18 years old, were pregnant, or had chronic obstructive pulmonary disease, recent arrhythmia; unstable angina or myocardial infarction; chest wall trauma; or high intracranial pressure. The Ethics Committee of Qilu Hospital, Shandong University, approved the study protocol and informed consent was obtained from the patient’s next of kin.

Each patient underwent insertion of a 4 F central venous catheter (ARROW Co., USA) in the subclavian vein with an injectate temperature-sensor housing (PV4046, Pulsion Medical Systems, Munich, Germany) and a 5 F femoral arterial catheter with a thermistor tip (PV2015L20, Pulsion Medical Systems, Munich, Germany). Both catheters were connected to the PiCCO *plus* system (Pulsion Medical Systems V7.1, Munich, Germany). Ventilation was maintained throughout the protocol.

### Quantitative CT analysis

Spiral CT of the thorax was performed from the apex to diaphragm (GE Lightspeed, GE Healthcare, USA), with continuous monitoring of heart rate, arterial blood pressure, and oxygen saturation. CT parameters were collimation scan, 20 mm; exposure, 120 kV and 220 mA; table speed, 25 mm/sec; and pitch, 1:1.

**Figure 1:**
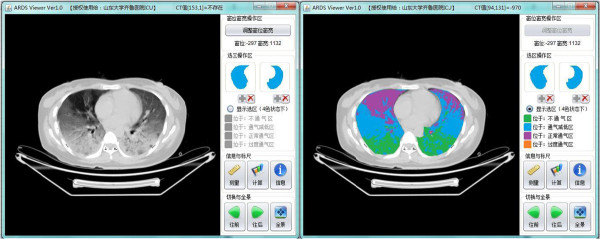
**Computed tomographic scans obtained in a 32-yr-old patient with “patchy” acute respiratory distress syndrome caused by invasive aspergillosis.** Contiguous 0.5-cm-thick computed tomographic sections were obtained from the apex to the diaphragm in positive end-expiratory pressure of 10 cmH_2_O. As shown by the ARDS viewer, the entire lung is composed of nonaerated (green), poorly aerated (blue), and normally aerated (purple) lung regions. The gas content (GV_CT_) is 506.86 ml, and the lung tissue volume (TV_CT_) is 1652.71 ml.

When patients were transported for CT scan, the ventilatory settings were maintained as in the ICU ward. The CT scan was performed quickly during an end-expiratory pause. Previous ventilatory settings were promptly reestablished at the end of the scan. CT images were analyzed by use of a home-made dedicated software, ARDS Viewer (Zhang Fan, Wu Dawei, Qilu Hospital, Shandong University, China). The ARDS Viewer allows for drawing the contour of the lung directly on a computer monitor (Figure 1). The mediastinum and pulmonary hila containing the trachea, main bronchi, and hilar blood vessels were excluded from the region of interest. Each element of the 512 × 512 matrix contained the value of the radiographic attenuation expressed in Hounsfield units (HU) corresponding to a volume of tissue (voxel).

Lung volume (gas + tissue) was calculated as the number of voxels present in a given lung region [5]. Despite the complex interface between gas (with radiologic density ~ -1000 HU) and tissue (radiologic density ~ 0 HU [water density]), one can compute the volume of gas, volume of tissue, overall lung volume, and volume distribution of lung aeration for any lung region [5,6]. The mean CT attenuation of a given lung region (CT lung density) is equivalent to its aeration: for example, -300 HU, the lung region is composed of 70% tissue and 30% gas (lung aeration = 30%). Therefore, the gas volume and tissue volume of each voxel can be calculated as follows [5,6]:

$$\begin{array}{ll} \mathrm{The}\;\mathrm{gas}\;\mathrm{volume} & \;=\frac{\mathrm{mean}\;\mathrm{CT}\;\mathrm{number}}{\mathrm{CT}\;{\mathrm{number}}_{\mathrm{gas}}‐\mathrm{CT}\;{\mathrm{number}}_{\mathrm{water}}}\times \;\mathrm{the}\;\mathrm{volume}\;\mathrm{of}\;a\;\mathrm{voxwel}\\ & \;=\frac{\mathrm{mean}\;\mathrm{CT}\;\mathrm{number}}{‐1000}\times \;\mathrm{the}\;\mathrm{volume}\;\mathrm{of}\;a\;\mathrm{voxwel}\\ \mathrm{The}\;\mathrm{gas}\;\mathrm{volume} & \;=\left(1‐\frac{\mathrm{mean}\;\mathrm{CT}\;\mathrm{number}}{‐1000}\right)\times \;\mathrm{the}\;\mathrm{volume}\;\mathrm{of}\;a\;\mathrm{voxwel} \end{array}$$

All voxels in the matrix have the same volume depending on the size of the pixels of the matrix and the thickness of the CT section (0.5 cm). Therefore, the volume of gas (GV_CT_) and volume of tissue (TV_CT_) of the overall lung can be obtained. The mean radiographic attenuation value (CTmean) for the whole lung was computed by averaging the attenuation value of all voxels in the region of interest. The gas-to-tissue ratio (g/t) was computed as the ratio between GV_CT_ and TV_CT_. The tissue volume index (TVI) was obtained by indexing TV_CT_ to predicted body weight of the patient.

**Figure 2:**
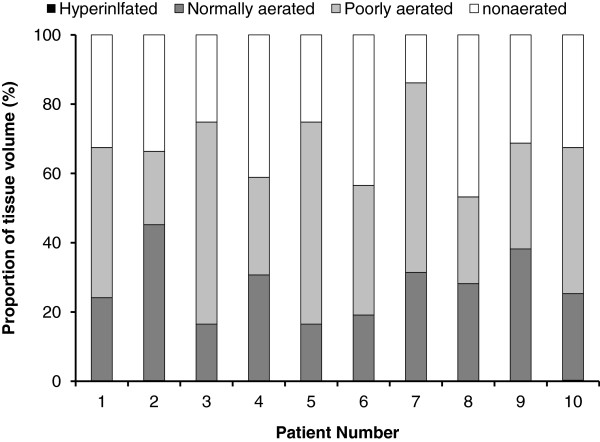
**Distribution of the tissue volume compartments for the whole lung in each patient.** Lung compartments were classified according to CT values as hyperinflated (CT values less than -900 HU), normally aerated (between -500 and -900 HU), poorly aerated (between -100 and -500 HU), and nonaerated (between -100 and + 100 HU).

Classically, lung aeration is quantified into 4 compartments [7-10]: 1) normal aeration, CT attenuation -900 to -500 HU, corresponding to a normal ventilation perfusion ratio; 2) overinflation, CT attenuation < -900 HU, corresponding to alveolar dead space if the lung region is insufficiently perfused; 3) poor aeration, CT attenuation -100 to -500 HU, which corresponds radiologically to “ground-glass” opacification or reticular pattern and physiologically to venous admixture if the lung region remains perfused; and 4) nonaeration, CT attenuation > -100 HU, corresponding radiologically to consolidation and atelectasis and physiologically to true pulmonary shunt if the lung region remains perfused. We computed the ratio for the tissue volumes of each compartment (Figure 2).

### Single-indicator thermodilution

The PiCCO *plus* system involves a thermal indicator to determine EVLW, cardiac output (CO), and volumetric measures. A central venous catheter and femoral arterial catheter with a thermistor tip are inserted for measurement. Cold saline (8°C), 15 ml volume, is injected into the central venous catheter. Then the thermistor tip on the femoral arterial catheter measures the downstream temperature change within the abdominal aorta. A transpulmonary thermodilution curve can be drawn. The cardiac output is calculated by the modified Stewart Hamilton equation from the area below the transpulmonary thermodilution curve. From the mean transit time (MTt) and the down slope time (DSt) of the exponential thermodilution curve, preload and lung water values are determined.

The intrathoracic thermal volume (ITTV) can be calculated by multiplying the CO by the MTt. The thermal indicator mixes with the largest possible volume of distribution.

ITTV=CO×MTt

The ITTV is composed of the pulmonary thermal volume (PTV) and the global end-diastolic volume (GEDV). PTV can be determined as the product of CO and the exponential DSt as follows.

$$\begin{array}{l} \mathrm{PTV}=\mathrm{CO}\times \mathrm{DSt}\\ \mathrm{GEDV}=\mathrm{ITTV}-\mathrm{PTV} \end{array}$$

Sakka and coworkers [29] examined the relationship between intrathoracic blood volume (ITBV) and GEDV and derived the following equation using linear regression to describe this relationship.

$$\mathrm{ITBV}=\left(1.25\times \mathrm{GEDV}\right)-28.4\;\mathrm{ml}.$$

EVLW reflects all fluid, including interstitial and alveolar, that is outside of the pulmonary vasculature during transit of the thermal indicator. EVLW is calculated as the difference between the ITTV and ITBV as follows.

EVLW=ITTV-ITBV

In the PiCCO *plus* system, ITBV and GEDV are indexed to the body surface area of each patient to obtain the intrathoracic blood volume index (ITBI) and global end-diastolic volume index (GEDI); EVLW is indexed to predicted body weight for the extravascular lung water index (ELWI). All values were obtained just after the patient returned to the ICU after CT scan.

### Other related values

Immediately after CT scan, arterial blood was drawn for blood gas analysis, PaO_2_/FiO_2_ was calculated_;_ and respiratory system compliance (Crs) was recorded. The Acute Physiology And Chronic Health Evaluation II (APACHE II) and Sequential Organ Failure Assessment (SOFA) scores were obtained from the worse record for each physiological variable within the 24 hr before and after CT scan.

### Statistical analysis

Data are presented as mean ± SD. To evaluate the association of PTV with TV_CT_ and ELWI with TVI, we performed a linear regression analysis. Agreement between values was assessed by the Bland and Altman plot method. Correlation was evaluated between the main values (PTV, ELWI, CTmean, GV_CT_, TVI, and g/t) and PaO_2_/FiO_2_, Crs, and APACHE II and SOFA scores. To assess whether pulmonary edema was associated with any specific tissue compartment, we correlated the ELWI with the relative tissue volume for each compartment. All correlations were assessed by linear regression analysis with the Pearson correlation coefficient *r*. Statistical significance was set at *P*<0.05. Statistical analyses involved use of SAS 9.2 (SAS Inst., Cary, NC).

## Results

### Patient characteristics

We included 10 patients (mean age 48.60 ± 18.08 years; 8 males); characteristics are in Table 1. Each patient showed a PaO_2_/FiO_2_ < 200 mmHg, with an overall mean 103.87 ± 54.42 mmHg. ARDS was due to pneumonia for 6 patients and multiple trauma for 2, with abdominal infection for 2. Half of the patients presented a large amount of pleural fluid.

**Table 1:** Clinical characteristics of patients

**Patient no.**	**Cause of ARDS**	**Sex**	**Age (yr)**	**PaO**_**2**_**/FiO**_**2**_**(mmHg)**	**Crs (ml/cmH**_**2**_**O)**	**APACHE II score**	**SOFA score**	**MVtime (day)**	**Pleural fluid**
1	Pneumonia	Male	60	58.00	13.24	19	9	4	No
2	Abdomen infection	Male	52	98.33	20.50	9	6	2	No
3	Pneumonia	Female	32	49.00	19.57	12	5	<1	No
4	Pneumonia	Male	58	75.41	30.00	11	6	<1	Yes
5	Multiple trauma	Male	56	98.36	34.90	21	7	2	Yes
6	Multiple trauma	Male	36	148.00	27.30	28	13	<1	Yes
7	Pneumonia	Female	19	100.00	37.50	10	10	4	No
8	Pneumonia	Male	78	185.00	30.00	14	6	5	Yes
9	Abdomen infection	Male	63	190.57	31.76	28	8	2	Yes
10	Pneumonia	Male	32	36.00	16.76	16	5	<1	No
Mean		8 Males/2 Females	48.6	103.87	26.15	16.80	7.50		
SD			18.08	54.42	8.15	7.04	2.55		

APACHE II: Acute Physiology And Chronic Health EvaluationII; ARDS, acute respiratory distress syndrome; Crs, respiratory system compliance; MVtime, time of mechanical ventilation before study enrolment; SOFA: Sequential Organ Failure Assessment.

### Quantitative CT analysis

On CT scan, TV_CT_ was 1221.37 ± 383.68 ml, TVI 18.12 ± 6.79 ml/kg, GV_CT_ 916.58 ± 418.08 ml, CTmean -407.97 ± 118.30 HU, and g/t 0.80 ± 0.40 (Table 2). Nonaerated, poorly aerated, normally aerated, and hyperinflated tissue represented 32.55 ± 9.26%, 39.92 ± 13.82%, 27.46 ± 9.29%, and 0.06 ± 0.11% of the TV_CT_, respectively (Figure 2).

**Table 2:** Main study variables for individual patients

**Patient no.**	**CT Quantitative analysis**					**PiCCO *plus *** **system**			
	**CT**_**mean**_**(Hu)**	**GV**_**CT **_**(ml)**	**TV**_**CT **_**(ml)**	**TVI (ml/kg)**	**g/t**	**GEDI (ml/m**^**2**^**)**	**ITBI (ml/m**^**2**^**)**	**PTV (ml)**	**ELWI (ml/kg)**
1	-408.76	1068.12	1519.39	17.67	0.70	916	1145	1580.9	13
2	-635.51	1253.14	712.60	10.96	1.76	853	1066	953.13	9
3	-240.55	506.86	1652.71	33.05	0.31	564	705	2404.6	44
4	-352.20	485.55	697.01	9.29	0.70	801	1001	891.38	7
5	-264.17	735.94	1422.71	19.76	0.52	904	1130	1565.6	16
6	-331.26	610.46	1209.24	15.50	0.50	920	1150	1614.2	15
7	-440.78	677.83	857.52	14.29	0.79	576	720	1007.4	13
8	-415.64	759.62	1044.63	17.41	0.73	901	1126	1564.9	20
9	-491.44	1296.89	1328.34	18.98	0.98	908	1135	1454.1	15
10	-499.44	1771.35	1769.50	24.24	1.00	580	725	1944.5	23
Mean	-407.97	916.58	1221.37	18.12	0.80	792.3	990.3	1498.1	17.5
SD	118.30	418.08	383.68	6.79	0.40	155.29	194.08	466.45	10.4

CTmean, mean CT values; GV_CT_, gas volume in lung measured by CT; TV_CT_, tissue volume in lung measured by CT; TVI, tissue volume index for predicted body weight; g/t, ratio of gas to tissue volume; GEDI, global end-diastolic volume index for patient surface area; ITBI, intrathoracic blood volume index for patient surface area; PTV, pulmonary thermal volume; ELWI, extravascular lung water index for predicted body weight.

### Single-indicator thermodilution

With the PiCCO *plus* system, PTV and ELWI were 1498.1 ± 466.45 mL and 17.5 ± 10.4 ml/kg, respectively. Individual values for GEDI, ITBI, PTV, and ELWI are in Table 2.

### Correlation between quantitative CT analysis and single-indicator thermodilution values

**Figure 3:**
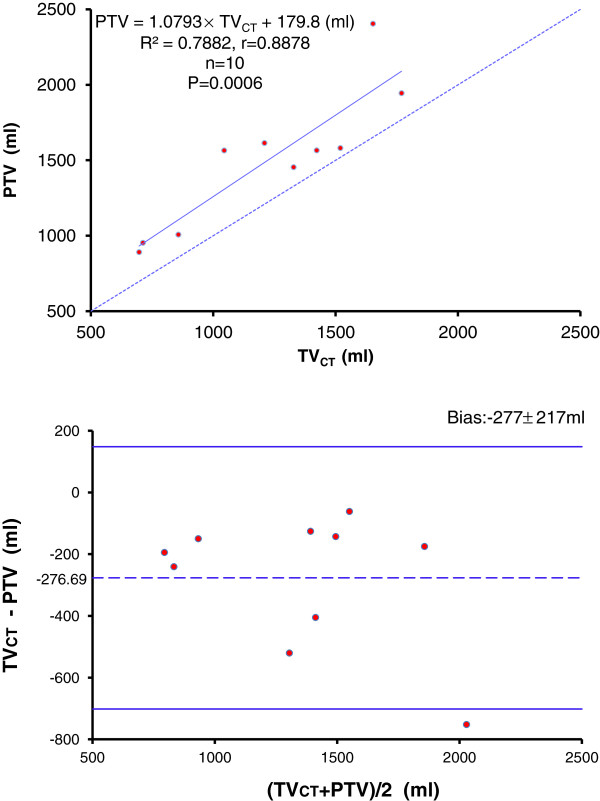
**Upper panel, correlation between TVCT and PTV.** Dashed line represents identity. Lower panel, Bland and Altman’s plot of agreement between TVCT and PTV: differences are plotted against average values. Mean difference (dashed line) and 95% confidence limit (bold lines) are indicated.

**Figure 4:**
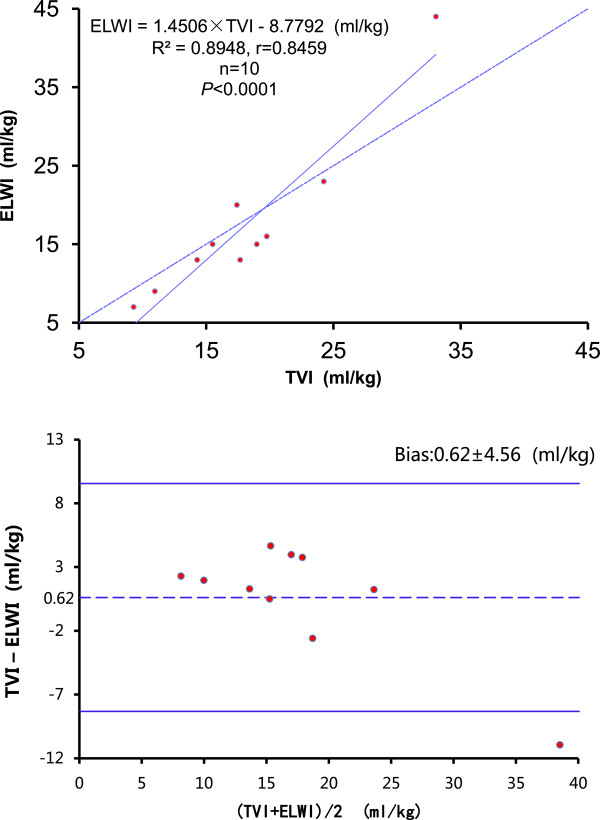
**Upper panel, correlation between TVI and ELWI.** Dashed line represents identity. Lower panel, Bland and Altman’s plot of agreement between TVI and ELWI: differences are plotted against average values. Mean difference (dashed line) and 95% confidence limit (bold lines) are indicated.

Mean TV_CT_ and PTV were positively correlated (*r* =0.8878; *P* = 0.0006; PTV = 1.0793 × TV_CT_ + 179.8) and PTV values overestimated those of TV_CT_ (Figure 3). Mean TVI and ELWI were positively correlated (*r* =0.9459; *P* < 0.0001; ELWI = 1.4506 × TVI - 8.7792). The agreement between TVI and ELWI was better than that for TV_CT_ and PTV, although mean TVI was slightly larger than ELWI (Figure 4). The bias between TV_CT_ and PTV, TVI and ELWI was -277 ± 217 and 0.62 ± 4.56, respectively.

We found no correlation between ELWI and proportion of any lung-tissue compartments. The differences between TVI and ELWI were not correlated with proportion of normally aerated, poorly aerated or nonaerated lung compartments.

### Pathophysiological correlations

We found no significant correlation between physiological variables (PaO_2_/FiO_2_, Crs, APACHE II and SOFA score) and CT or single-indicator thermodilution variables (Table 3).

**Table 3:** Correlation coefficients for CT or PiCCO variables with other parameters

**Variables**	**CT Quantitative analysis**				**PiCCO *plus *** **system**	
	**CTmean**	**GV**_**CT**_	**TVI**	**g/t**	**PTV**	**ELWI**
PaO_2_/FiO_2_	-0.10	0.03	-0.45	0.08	-0.39	-0.14
Crs	0.01	-0.24	-0.50	-0.01	-0.54	-0.18
APACHE II score	0.28	0.21	0.50	-0.30	0.47	0.35
SOFAscore	0.08	-0.12	-0.26	-0.13	-0.18	-0.43

CTmean, mean CT values; GV_CT_, volume of gas in lungs measured by CT; TVI, tissue volume index for predicted body weight; g/t, ratio of gas to tissue volume; PTV, pulmonary thermal volume; ELWI, extravascular lung water index for predicted body weight; Crs, compliance of respiratory system; APACHE II: Acute Physiology And Chronic Health Evaluation II; SOFA: Sequential Organ Failure Assessment.

## Discussion

In patients with ARDS, lung tissue content measured by quantitative CT analysis was highly correlated with ELWI and PTV measured by the single-indicator thermodilution method.

As a convenient, minimally invasive, and economical technique, PiCCO monitoring has been generally used in ICUs all over the world. As compared with the gravimetric method, single-indicator thermodilution to determine EVLW shows good correlation in animal models of lung injury, although EVLW is slightly higher with the latter method [23-25]. Tagami and colleagues found that measurement of EVLW by the single-transpulmonary system is closely correlated with post-mortem lung weight in humans [26]. Overestimation of EVLW may have several explanations. First, the thermal indicator is also equilibrated with the myocardium and vessel walls, which may lead to a large volume of distribution and a small increase in EVLW measurement. Second, recirculation of cold saline can overestimate lung water [23]. Third, the linear regression equations between ITBV and GEDV may differ among species [25,30].

An animal experiment indicated that as compared with postmortem gravimetry, TVI estimated by quantitative CT had acceptable accuracy in tracking the extent of pulmonary edema, with a mean bias of 4 ml/kg [27]. Recent experimental and clinical investigations have revealed that CT-derived pulmonary tissue volume is closely associated with EVLW and PTV determined by thermal indocyanine green-dye double-indicator dilution, but TV_CT_ showed a tendency to be larger than PTV or EVLW [27,28]. This overestimation might be due to the additive effects of several factors. First, CT does not differentiate between lung compartments with interstitial fluid, pulmonary tissue, or residual intra-vascular blood. Second, CT reveals fluids in the alveoli and pleural space that might escape detection with indicator dilution methods.

Many factors may limit the reliability of EVLW determined by single-indicator thermodilution. First, the relationship between GEDV and ITBV may be affected by anatomy (height, weight), mechanics (tidal volume, PEEP), physiologic features (pulmonary edema, hypoxic vasoconstriction), and pharmacologic features (vasoactive drugs) [13,31]. The equation between GEDV and ITBV differs among species [25,30]. So EVLW may be affected by all of these factors. Second, the thermodilution method for measuring EVLW relies on heat exchange across the alveolar epithelial and endothelial barriers. If the thermal indicator does not have access to all lung tissue because of decreased perfusion, then EVLW will be consistently underestimated. Several pathophysiological changes in critically ill patients, such as large pulmonary vessel obstruction, pulmonary vascular microembolism, and hypoxic vasoconstriction, can impair the perfusion and result in an inaccurate EVLW [14,15]. Easley and co-workers found that inactivating hypoxic pulmonary vasoconstriction by endotoxin administration increased pulmonary blood flow to poorly aerated regions, with increased EVLW [32]. Third, PEEP may affect the measured value of EVLW and directly affect the amount of lung water present, thereby indirectly affecting EVLW. High levels of PEEP may lead to pulmonary capillary collapse, thus potentially resulting in underestimating EVLW. However, the use of PEEP may cause a redistribution of pulmonary blood flow toward previously underperfused lung regions, thus leading to a more accurate EVLW. Several trials have given different results for the influence of PEEP on measurement of EVLW [18-21]. Fourth, the degree of lung edema can affect the accuracy of EVLW. However, the exact influence is still controversial [13,21,22]. Finally, many anatomic and physiological abnormalities that can alter EVLW include major pulmonary resection, the presence of large aortic aneurysms, arterial catheters placed too far peripherally, and intracardiac shunts [29].

The results of this study indicate that in patients with ARDS, EVLW and PTV measured by the single-indicator thermodilution method were highly correlated with lung tissue content measured by CT. However, EVLW and PTV determined with single-indicator thermodilution overestimated TV_CT_ perhaps because CT cannot differentiate between lung compartments with interstitial fluid, pulmonary tissue, or residual intra-vascular blood. As well, other factors that can limit the reliability of EVLW measured by single-indicator thermodilution include the level of PEEP, the extent of the shunt in the lungs, and recirculation of cold saline. One trail estimated the CT-based ELWI by two different radiologists without analyzing software [33]. CT-based estimation of ELWI is not accurate for predicting extravascular lung water in critically ill patients when compared to single-indicator thermodilution, because they only use some CT findings to guess the ELWI [33]. But in our study, the quantitative CT analysis is much more accurate. The presence of a large amount of pleural fluid in 5 patients may have affected the reliability of EVLW obtained by single-indicator thermodilution. Two previous studies revealed that fluid in the pleural space did not contribute to the measurement of EVLW [34,35]. However, one of the studies indicated that pleural fluid contributed to increased PTV [34]. Saugel and coworkers recently found that large-volume thoracentesis significantly increased EVLI measured by single-indicator thermodilution [36]. However, EVLI was higher after removal of pleural fluid; the authors concluded that pleural effusions did not take part in single-indicator transpulmonary thermodilution as part of the pulmonary thermovolume and did not increase EVLI [36].

ELWI and the differences between TVI and ELWI were not correlated with proportions of any lung compartments. This result agreed with Patroniti and coworkers [28], but we lack other data for comparison.

We found no correlation between CT or PiCCO variables and Crs or PaO_2_/FiO_2_. This result agreed with some previous research [26,37,38], although it still remains controversial. PaO_2_/FiO_2_ was affected by many factors, such as PEEP and inhaled oxygen concentration. Even for the same patient, the position, sedation state, and airway secretions can lead to changes in PaO_2_/FiO_2_[39]. Dynamic changes in EVLW showed a significant negative correlation with PaO2/FiO2 and Crs changes [40], which suggests that the dynamic monitoring of EVLW is important for the assessment of severity and treatment effects.

APACHE II and SOFA scores have been widely used to assess the severity of critically ill patients and were closely related to the prognosis of multiple organ dysfunction syndrome [41,42]. We found no correlation between ELWI or TVI and these scores; APACHE II and SOFA scores may not be specific markers of acute lung injury but they can help in assessing the overall condition of patients.

Because of the small population and the lack of previous data to compare with our findings, further studies are necessary to confirm our results. As well, the time of mechanical ventilation before enrollment and ventilator settings were not the same for each patient, which may have affected our results.

## Conclusions

Measuring pulmonary edema by quantiative CT analysis shows good agreement with that by single-indicator thermodilution.

### Key messages

• Interstitial lung edema is the most important pathological character of acute respiratory distress syndrome. Quantitative measurement of lung edema can provide a useful marker of disease severity and prognosis.

• Measurement of pulmonary edema by quantitative CT analysis showed good agreement with that by single-indicator thermodilution, although extravascular lung water and pulmonary thermal volume values were underestimated by the latter method for tissue volume of the lung.

• As a noninvasive method, quantitative CT analysis is a gold standard for evaluating lung aeration, which may have clinical relevance in the mechanical ventilation setting and provide a quantitative “in vivo” measurement to estimate pulmonary edema.

## Abbreviations

CT: Computed tomography; ALI/ARDS: Acute lung injury/acute respiratory distress syndrome; APACHE II: Acute Physiology And Chronic Health Evaluation II; CO: Cardiac output; Crs: Compliance of respiratory system; Crs: Compliance of respiratory system; CTmean: Mean radiographic attenuation value for the whole lung; g/t: Gas-to-tissue ratio; DSt: Exponential downslope decay time; EVLW: Extravascular lung water; ELWI: Extravascular lung water index for predicted body weight; ITBI: Intrathoracic blood volume index for patient surface area; ITTV: Intrathoracic thermal volume; ITBV: Intrathoracic blood volume; GEDI: Global end-diastolic volume index for patient surface area; GEDV: Global end-diastolic volume; g/t: Ratio of gas to tissue volume; GVCT: Quantitative CT analysis of gas volume; MODS: Multiple organ dysfunction syndrome; MTt: Mean transit time; PEEP: Positive end-expiratory pressure; PTV: Pulmonary thermal volume; PBV: Pulmonary blood volume; SOFA: Sequential Organ Failure Assessment; TVI: Tissue volume index for predicted body weight; TVCT: Quantitative CT analysis of tissue volume.

## Competing interests

The authors declare that they have no competing interests.

## Authors’ contributions

FZ, CL and DW designed the study. FZ, CL and JZ planned and collected the data. FZ, CL and HG wrote the manuscript. All authors read and approved the final manuscript.
